# Predictive value of primary tumor ^18^F-FDG PET/CT metabolic parameters for lymph node metastasis in treatment-naïve ALK-positive NSCLC

**DOI:** 10.1186/s40644-026-01058-0

**Published:** 2026-05-29

**Authors:** Tingting Qiao, Jie Gu, Yangchun Chen, Juan Zhao

**Affiliations:** Department of Nuclear Medicine, Shanghai Pulmonary Hospital, Tongji University School of Medicine, 507 Zhengmin Road, Shanghai, 200433 China

**Keywords:** ^18^F-FDG PET/CT, ALK, NSCLC, Lymph node metastasis, SUVmax

## Abstract

**Purpose:**

To investigate the association between primary tumor metabolic parameters on ^18^F-FDG PET/CT and lymph node metastasis (LNM) in treatment-naïve anaplastic lymphoma kinase (ALK)-positive non-small cell lung cancer (NSCLC), and to evaluate their utility in discriminating the cases with radiographically occult LNM (OLNM).

**Methods:**

We retrospectively analyzed 157 patients with pathologically confirmed ALK-positive NSCLC who underwent ^18^F-FDG PET/CT before the treatment between October 2021 and December 2024. Primary tumor metabolic parameters, including SUVmax, SUVpeak, metabolic tumor volume (MTV), and total lesion glycolysis (TLG), were measured. Logistic regression analyses were performed to identify LNM predictors. Receiver operating characteristic curve analysis evaluated predictive performance, with area under the curve (AUC) was calculated. The relation between OLNM status and metabolic parameters of primary tumor was analyzed using Kruskal-Wallis test.

**Results:**

Among 157 patients (62 males and 95 females; mean age 56 ± 11 years), 71 patients (45.2%) had pathologically confirmed LNM. Patients with LNM exhibited evidently larger primary tumor size and higher SUVmax, SUVpeak, MTV, and TLG than those without LNM (all *P* < 0.001). Univariate analysis showed primary tumor size, SUVmax, SUVpeak, MTV, and TLG were remarkedly associated with LNM. Multivariate analysis identified primary tumor size (OR: 1.043, 95%CI: 1.004–1.084; *P* = 0.032) and SUVmax (OR: 1.322, 95%CI: 1.159–1.532; *P* < 0.001) as independent predictors. The AUCs for tumor size, SUVmax, and combined factors were 0.76, 0.77, and 0.81, respectively. In addition, false-negative (OLNM-positive) cases showed markedly larger tumor size (*P* = 0.003) and higher TLG (*P* = 0.011) than true-negative cases.

**Conclusion:**

Metabolic parameters of ^18^F-FDG PET/CT before treatment, particularly when combined with primary tumor size, may help stratify LNM risk in treatment-naïve ALK-positive NSCLC patients. These parameters may help identify OLNM, potentially guiding personalized lymph node dissection strategies and optimizing treatment plans.

## Introduction

Anaplastic lymphoma kinase (ALK) gene rearrangement defines a distinct molecular sub-type of non-small cell lung cancer (NSCLC) characterized by unique biological behavior and clinical implications [1, 2]. ALK-positive NSCLC is predominantly driven by chromosomal fusions involving echinoderm microtubule-associated protein-like 4, which promotes tumor proliferation, survival, and metastasis [3, 4]. This molecular alteration increases the propensity of tumor metastasis, particularly to lymph nodes, which may also be associated with radiographically occult lymph node metastasis (OLNM) [5–7]. Patients with ALK-positive NSCLC show significant sensitivity and high objective response to ALK tyrosine kinase inhibitors (TKIs) [8–11], establishing this cohort as prime candidates for precision therapy. Lymph node metastasis (LNM) serves as a critical independent prognostic factor in NSCLC, with a distinct difference in 5-year survival rates between patients with and without LNM [12–14]. In ALK-positive NSCLC, LNM status directly influences lymph node dissection strategies and ALK-TKI treatment planning. However, despite advances in preoperative staging, unexpected nodal metastases are frequently detected post-surgery, highlighting the urgent need for novel predictive biomarkers of LNM in this population.

^18^F-fluorodeoxyglucose positron emission tomography/computed tomography (^18^F-FDG PET/CT) has emerged as a pivotal tool in tumor staging, offering superior diagnostic accuracy over conventional CT by integrating metabolic and anatomical data [15–17]. Semi-quantitative PET/CT parameters, such as maximum standardized uptake value (SUVmax), maximum average SUV within a 1-cm^3^ spherical volume (SUVpeak), metabolic tumor volume (MTV), and total lesion glycolysis (TLG), reflect tumor glucose metabolism and have demonstrated prognostic value in NSCLC [18–20], including prediction of LNM [21, 22]. However, these findings derived from general NSCLC populations may not be directly translatable to the ALK-positive subgroup, which is known for its more aggressive biology and higher propensity for LNM.

While ^18^F-FDG PET/CT parameters have been extensively studied in general NSCLC populations, the utility of ^18^F-FDG PET/CT metabolic parameters for predicting LNM, especially radiographically OLNM, in ALK-positive NSCLC cohort, remains underexplored. Given the aggressive nodal metastatic tendency of ALK-positive NSCLC patients, we hypothesized that primary tumor metabolic features may correlate with LNM risk. Therefore, this study was designed to investigate the association between primary tumor metabolic parameters (SUVmax, SUVpeak, MTV, and TLG) on ^18^F-FDG PET/CT and LNM status in patients with treatment-naïve ALK-positive NSCLC. We aim to fill this critical knowledge gap, thereby optimizing individualized lymph node dissection strategies and improving prognostic stratification.

## Materials and methods

### Patient selection

We retrospectively enrolled 157 patients with pathologically confirmed ALK-positive NSCLC at Shanghai Pulmonary Hospital between October 2021 and December 2024. All patients underwent complete surgical resection of the primary tumor combined with systematic lymph node dissection, and met the following inclusion criteria: (1) No prior systemic or local anticancer therapy; (2) Absence of concurrent malignancies or history of other cancers; and (3) ^18^F-FDG PET/CT performed within 2 weeks before tissue sampling. This study was approved by the Institutional Review Board of Shanghai Pulmonary Hospital as a retrospective study; thus, the requirement for patient informed consent was waived. The research protocol adhered to the principles of the Declaration of Helsinki.

### ^18^F-FDG PET/CT imaging protocol

PET/CT scans were performed on a Biograph 64 system (Siemens Healthineers, Erlangen, Germany) with a 21.6-cm axial field of view. Patients fasted for ≥ 6 h pre-scan, with serum glucose levels maintained at < 7.4 mmol/L. After intravenous injection of 3.7 MBq/kg (maximum dose: 370 MBq) of ^18^F-FDG, image acquisition commenced at 60 ± 5 min post-injection. Emission data were acquired from the skull base to mid-thigh using 6–7 bed positions (2.5 min/bed position). Non-contrast CT was performed for attenuation correction and anatomical localization (120 kV; auto mA modulation with reference 101 mA; 5-mm slice thickness). PET images were reconstructed using ordered-subset expectation maximization (3 iterations, 21 subsets) with CT-based attenuation correction, applying a 5.0 mm FWHM Gaussian filter. All reconstructions utilized a 200 × 200 matrix.

### ^18^F-FDG PET/CT metabolic parameters analysis

Two experienced nuclear medicine physicians independently reviewed all PET/CT images. Both reviewers were blinded to all pathological data, including the final ALK status and nodal stage. Any discrepancies in interpretation were resolved by consensus. Metabolic parameters of the primary tumor, including SUVmax, SUVpeak, MTV and TLG, were quantified using syngo.via workstation (Siemens Healthineers, Erlangen, Germany). MTV (cm³) was defined as the volume segmented by a 40% SUVmax threshold. TLG was calculated as: TLG = MTV × SUVmean. The tumor size is the maximum diameter of the primary tumor. To evaluate the inter-observer reproducibility of the quantitative measurements, the intraclass correlation coefficient (ICC) with a two-way random-effects model for absolute agreement was calculated for each metabolic parameter (SUVmax, SUVpeak, MTV, TLG) based on the independent assessments prior to consensus.

### Histopathological and genetic analyses

All patients underwent tumor resection within 2 weeks post ^18^F-FDG PET/CT. LNM status for all patients was definitively determined by histopathological examination of the systematically dissected lymph node stations obtained during surgery. OLNM was defined as pathologically confirmed metastasis in ^18^F-FDG PET/CT-negative nodes. Hematoxylin-eosin and immunohistochemical staining were performed for tumor typing. ALK-rearrangement was detected using the Amplification Refractory Mutation System PCR (ARMS-PCR; Amoy Diagnostics, Xiamen, China) with β-actin as the internal control. Reactions included positive (ALK-rearranged cell line) and negative (no-template) controls, and results were interpreted per manufacturer thresholds. All histopathological and genetic findings were independently reviewed by two board-certified pathologists, with disagreement resolved by consensus.

### Statistical analysis

Statistical analyses were performed using SPSS 26.0 (IBM Corp., Armonk, NY, USA). Normally distributed data are expressed as mean ± standard deviation (SD), while non-normally distributed data are presented as median (interquartile range, Q1–Q3). Categorical variables are summarized as counts (percentages). Group comparisons for continuous variables utilized the independent-sample t-test (normal distribution) or Mann-Whitney U test (non-normal distribution). The inter-observer agreement for the continuous metabolic parameters was assessed using ICC analysis. ICC values range from 0 to 1, with higher values indicating greater reliability. ICC value greater than 0.75 was considered indicative of good agreement. For multi-group comparisons, the Kruskal-Wallis test was applied, with Bonferroni correction for pairwise comparisons. Categorical variables were compared using the chi-square test. Univariate and multivariate logistic regression analyses identified independent predictors of LNM. Diagnostic performance of metabolic parameters was evaluated using receiver operating characteristic (ROC) curve analysis, with area under the curve (AUC) with 95% confidence interval (CI) was calculated. Statistical significance was defined as *P* < 0.05.

## Results

### Association between LNM status and metabolic parameters of primary tumor in ALK-positive NSCLC

A total of 157 patients with ALK-positive NSCLC were enrolled in the present study. We found that all patients with ALK-positive NSCLC were adenocarcinoma patients in this study. Among these ALK-positive ADC patients, 71 (45.2%) patients were clinically diagnosed with pathologically proven LNM as the LNM group, while 86 (54.8%) patients were diagnosed with non-LNM as the NLNM group. All 157 cases included 95 females (60.5%) and 62 males (39.5%), with an average age of 56 ± 11 years (range, 32–77 years).

Excellent inter-observer agreement was found between the two physicians for the measurement of metabolic parameters, with ICCs of 0.98 for SUVmax, 0.98 for SUVpeak, 0.97 for MTV, and 0.97 for TLG.

As detailed in Table 1, patients with LNM exhibited elevated primary tumor metabolic parameters and larger tumor size compared to the NLNM group. Specifically, patients with LNM presented with a larger tumor size [27.0 (IQR 20.0–41.0) vs. 16.5 (13.0-23.2)] and higher metabolic values, including SUVmax [8.9 (6.0-12.4) vs. 5.0 (3.6–6.9)], SUVpeak [6.6 (4.2–9.6) vs. 3.3 (2.2-5.0)], MTV [2.5 (0.9–7.2) vs. 0.7 (0.3–1.8)], and TLG [13.4 (4.5–48.1) vs. 2.3 (0.8–5.8)] (all *P* < 0.001).

**Table 1:** Clinical information and ^18^F-FDG PET/CT metabolic parameters in treatment-naïve ALK-positive NSCLC patients

Clinical characteristic		All	LNM	NLNM	*P* value
Number (% of total)		157	71 (45.2)	86 (54.8)	
Age (y)					0.38
	Mean ± SD	56 ± 11	54.7 ± 10.1	56.7 ± 11.1	
	Range (years)	32–77	35–75	32–77	
BMI					0.68
	Mean ± SD	23.9 ± 3.21	23.8 ± 3.19	24.0 ± 3.24	
Gender, n (%)					0.75
	Female	95 (60.5%)	42(26.8%)	53 (33.8%)	
	Male	62 (39.5%)	29(18.5%)	33 (21%)	
Smoking history, n (%)					0.84
	Smoker	43(27.4%)	20(12.7%)	23(14.6%)	
	Never smoker	114(72.6%)	51(32.5%)	63(40.1%)	
Primary tumor location					0.84
	Left	81(51.6%)	36(22.9%)	45(28.7%)	
	Right	76(48.4%)	35(22.3%)	41(26.1%)	
Primary tumor size (mm)					< 0.001
	Median (Q1, Q3)	21.0(14.5–31.0)	27.0(20–41.0)	16.5(13.0-23.2)	
SUVmax					< 0.001
	Median (Q1, Q3)	6.4(4.1–9.4)	8.9(6.0-12.4)	5.0(3.6–6.9)	
SUVpeak					< 0.001
	Median (Q1, Q3)	4.3(2.8–6.7)	6.6(4.2–9.6)	3.3(2.2-5.0)	
MTV					< 0.001
	Median(Q1, Q3)	1.1(0.4–3.2)	2.5(0.9–7.2)	0.7(0.3–1.8)	
TLG					< 0.001
	Median(Q1, Q3)	4.7(1.4–18.3)	13.4(4.5–48.1)	2.3(0.8–5.8)	

Abbreviations: LNM, lymph node metastasis; NLNM, non-lymph node metastasis; NSCLC, non-small-cell lung cancer; BMI, body mass index; SUVmax, maximum standardized uptake value; SUVpeak, peak standardized uptake value; TLG, total lesion glycolysis; MTV, metabolic tumor volume, SD, standard deviation

In this study, no significant differences were observed between the LNM and NLNM groups regarding age, gender, BMI, smoking history, and primary tumor location in ALK-positive NSCLC (all *P* > 0.05).

### Univariate and multivariate analyses for LNM status in ALK-positive NSCLC

In patients with ALK-positive NSCLC, univariate logistic regression analysis initially identified several metabolic parameters and tumor size as being significantly associated with the presence of LNM. These included primary tumor size (OR: 1.087, 95%CI: 1.051–1.125; *P* < 0.001), SUVmax (OR: 1.410, 95%CI: 1.246–1.595; *P* < 0.001), SUVpeak (OR: 1.584, 95%CI: 1.347–1.862; *P* < 0.001), MTV (OR: 1.190, 95%CI: 1.071–1.322; *P* < 0.001), and TLG (OR: 1.035, 95%CI: 1.015–1.055; *P* < 0.001) (Table 2).

**Table 2:** Univariate and multivariate analysis of various factors association with LNM status in treatment-naïve ALK-positive NSCLC patients

Characteristics		Univariate analysis OR (95% CI)	*P* value	Multivariate analysis OR (95% CI)	*P* value
Age		0.983 (0.954–1.013)	0.254		
BMI		0.980 (0.888–1.081)	0.680		
Gender			0.752		
	Male	Reference			
	Female	0.902 (0.474–1.714)			
Smoking status			0.842		
	Never smoker	Reference			
	Ever smoker	1.074 (0.531–2.171)			
Primary tumor location			0.840		
	Left	Reference			
	Right	1.067 (0.569–2.001)			
Primary tumor size		1.087 (1.051–1.125)	< 0.001	1.043 (1.004–1.084)	0.032
SUVmax		1.410 (1.246–1.595)	< 0.001	1.322 (1.159–1.532)	< 0.001
SUVpeak		1.584 (1.347–1.862)	< 0.001		
MTV		1.190 (1.071–1.322)	< 0.001		
TLG		1.035 (1.015–1.055)	< 0.001		

Abbreviations: SUVmax, maximal standard uptake value; SUVpeak, peak standardized uptake value; MTV, metabolic tumor volume; TLG, total lesion glycolysis; OR, odds ratio; CI, confidence interval

To further evaluate the diagnostic performance of these significant parameters, ROC analysis was conducted. The performance results are summarized in Table 3. All metabolic parameters demonstrated good predictive value for LNM. Specifically, the AUC for primary tumor size was 0.76 (95% CI: 0.69–0.84) with an optimal cut-off of 25.5. Among the metabolic parameters, SUVmax showed an AUC of 0.77 (95% CI: 0.70–0.85) at a cut-off of 8.08, while SUVpeak and TLG both achieved the highest AUC of 0.78 (SUVpeak 95% CI: 0.71–0.86, cut-off = 6.51; TLG 95% CI: 0.71–0.85, cut-off = 5.94). And MTV had an AUC of 0.75 (95% CI: 0.68–0.83, cut-off = 2.29). Notably, TLG provided the highest sensitivity (70%), while SUVpeak offered the highest specificity (94%).

**Table 3:** Differential diagnostic efficacies of the ^18^F-FDG PET parameters for predicting LNM in treatment-naïve ALK-positive NSCLC patients

Parameter	Cut-off	AUC (95% CI)	Sensitivity	Specificity
Tumor size	25.5	0.76(0.69–0.84)	56%	83%
SUVmax	8.08	0.77(0.70–0.85)	56%	86%
SUVpeak	6.51	0.78(0.71–0.86)	54%	94%
TLG	5.94	0.78(0.71–0.85)	70%	77%
MTV	2.29	0.75(0.68–0.83)	56%	84%

Abbreviations: SUVmax, maximal standard uptake value; SUVpeak, peak standardized uptake value; MTV, metabolic tumor volume; TLG, total lesion glycolysis; CI, confidence interval

**Fig. 1:**
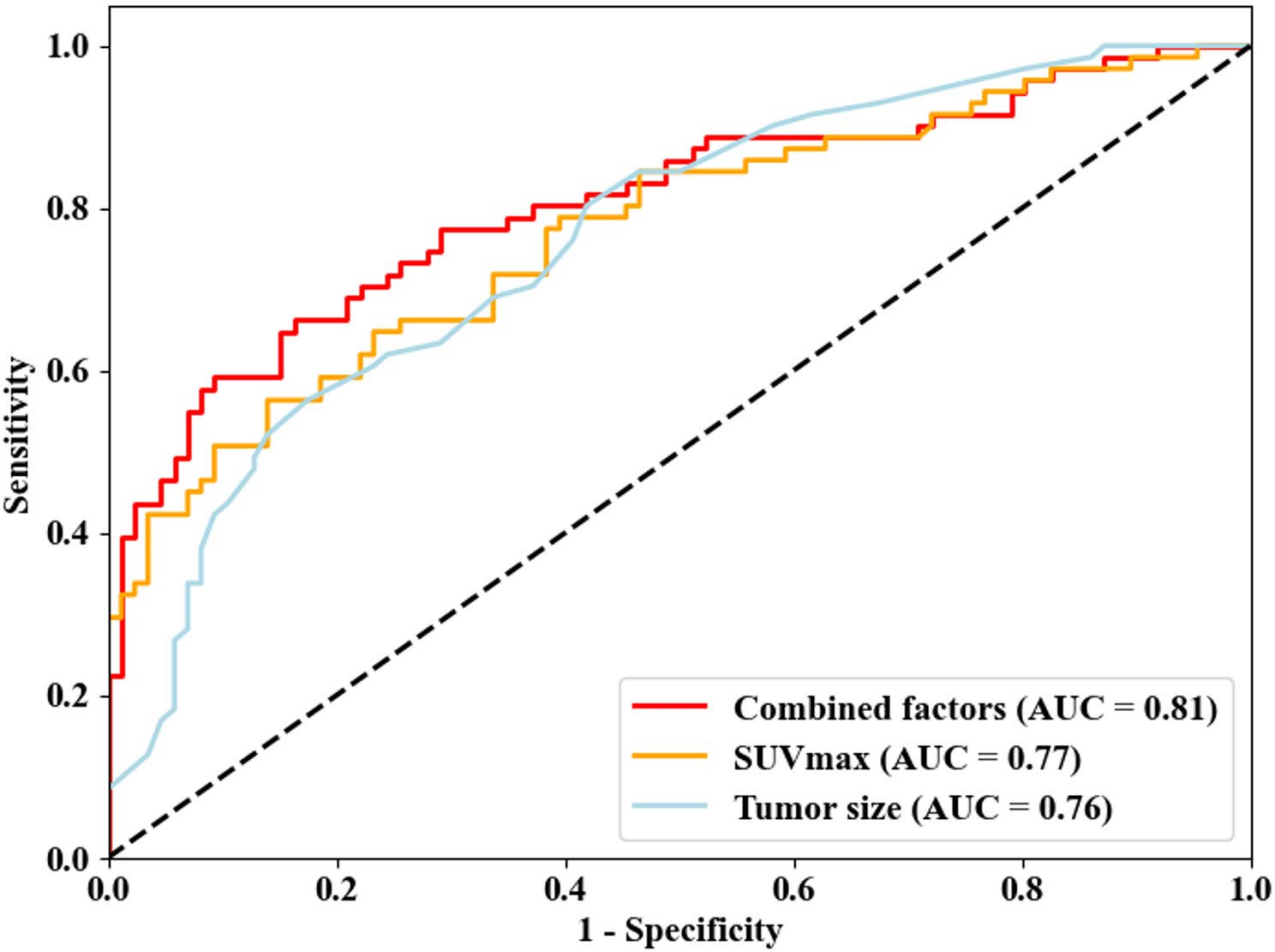
ROC curves for predicting LNM status in treatment-naïve ALK-positive NSCLC patients. SUVmax, maximum standardized uptake value; AUC, area under the curve

Subsequently, multivariate logistic regression analysis was performed to identify independent predictors. After adjusting for these factors, only larger tumor size (OR: 1.043, 95%CI: 1.004–1.084; *P* = 0.032) and higher SUVmax (OR: 1.322, 95%CI: 1.159–1.532; *P* < 0.001) remained as independent predictors of LNM (Table 2). Finally, a combined predictive model was constructed, which achieved an improved AUC of 0.81 (95% CI: 0.74–0.88) (Fig. 1).

## Association between OLNM status and metabolic parameters of primary tumor in ALK-positive NSCLC

Among 71 ALK-positive NSCLC patients with LNM, ^18^F-FDG PET/CT only detected 46 cases of LNM, while 25 patients were false-negative cases, indicating OLNM. The sensitivity and negative predictive value (NPV) of ^18^F-FDG PET/CT for detecting LNM in these ALK-positive NSCLC patients were 64.8% and 76.9%, respectively, while the specificity and positive predictive value (PPV) were 96.5% and 93.9%, respectively (Table 4). Therefore, relying solely on the assessment of lymph node status in lymph nodal regions by ^18^F-FDG PET/CT is insufficient for assessing OLNM. We propose that ^18^F-FDG PET/CT parameters of the primary tumor may improve the diagnostic performance of OLNM evaluation. In this study, we further analyzed differences in primary tumor ^18^F-FDG PET/CT parameters (SUVmax, SUVpeak, TLG, and MTV) and tumor size among the false-negative (OLNM) group, true-negative (TN) group, and true-positive (TP) group. Due to the low number of false-positive cases (*n* = 3), this group was excluded from the analysis.

**Table 4:** Diagnostic performance of ^18^F-FDG PET/CT in detecting LNM in treatment-naïve ALK-positive NSCLC patients

	TP	TN	FP	FN	Sensitivity	Specificity	NPV	PPV
^18^F-FDG PET/CT	46	83	3	25	64.79%	96.51%	76.9%	93.9%

Abbreviations: FN, false-negative; TN, true-negative; TP, true-positive; FP, false-positive; NPV, negative predictive value; PPV, positive predictive value

**Fig. 2:**
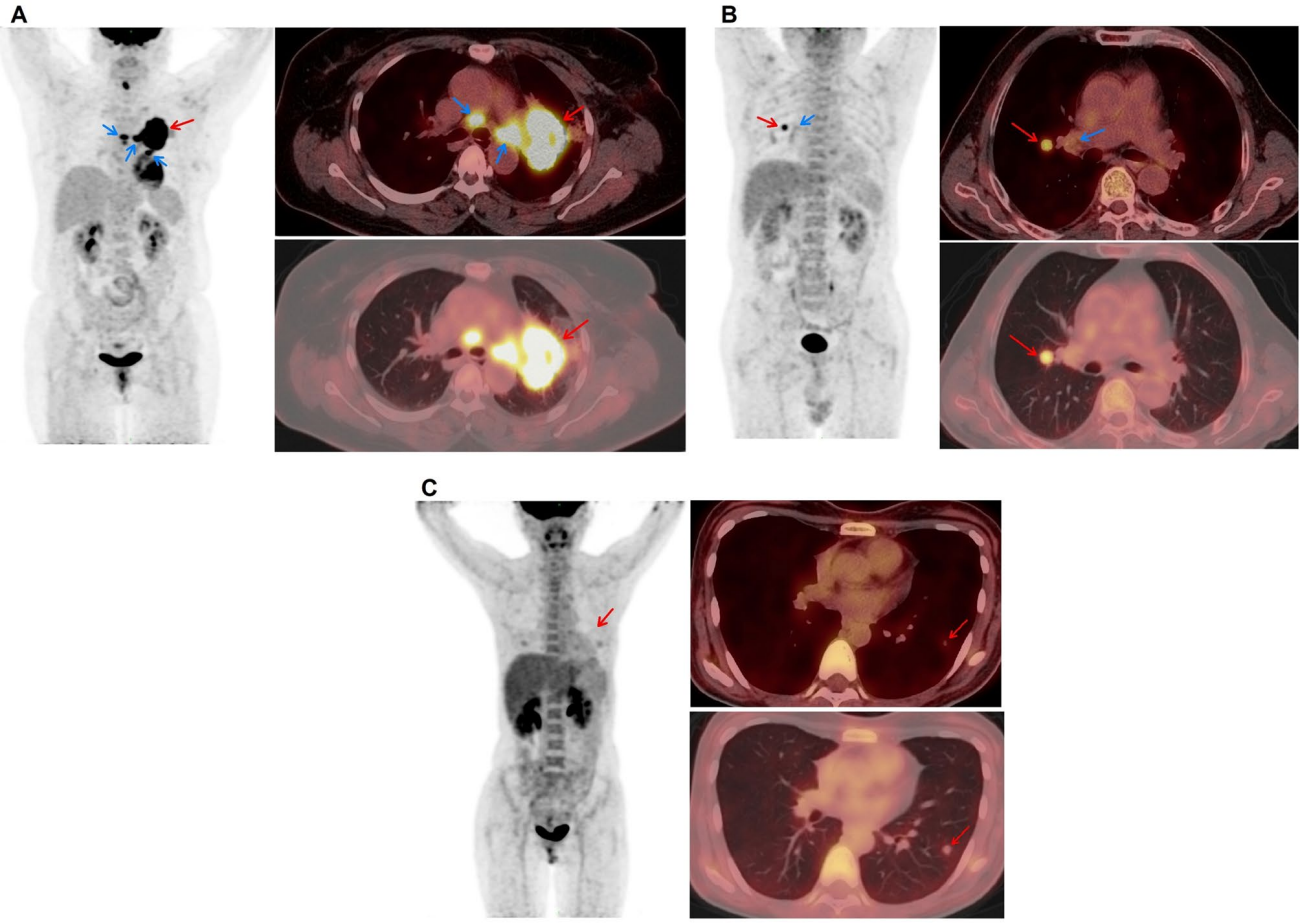
(**A)** A representative case of a patient with PET/CT-positive lymph nodes and pathologically confirmed LNM (true-positive). (**B**) A representative case of radiographically occult LNM (OLNM), where lymph nodes were PET/CT-negative but pathologically proven to harbor metastasis (false-negative). (**C**) A representative case of a patient with PET/CT-negative lymph nodes and pathologically confirmed absence of LNM (true-negative). The red arrows point to the primary tumor, and the blue arrows point to the metastatic lymph nodes

The Kruskal-Wallis test revealed significant differences in SUVmax, SUVpeak, TLG, MTV, and tumor size among the three groups (all *P* < 0.05; Table 5). Pairwise post hoc comparisons of the three groups demonstrated that the TP group exhibited significantly higher SUVmax, SUVpeak, TLG, MTV, and tumor size than the false-negative (OLNM) and the TN group (all *P* < 0.05). However, post hoc comparisons showed statistical differences between the false-negative (OLNM) group and the TN group only in terms of primary tumor size (*P* = 0.031) and TLG (*P* = 0.044). The primary tumor size and TLG of false-negative (OLNM) group were markedly higher than those of TN group. No significant differences were observed between the TN and OLNM groups for SUVmax (*P* = 0.101), SUVpeak (*P* = 0.118), or MTV (*P* = 0.066). To clearly and intuitively display the primary tumor imaging corresponding to the different lymph node states (TN, TP, false negative/OLNM) mentioned above, we have added typical ¹⁸F-FDG PET/CT images as Fig. 2.

**Table 5:** Correlation of primary tumor ^18^F-FDG PET/CT parameters with TP, TN, and FN status in treatment-naïve ALK-positive NSCLC patients

^18^F-FDG PET parameter		TN	TP	FN	*P* value
		83	46	25	
SUVmax	Median (Q1, Q3)	5.01 (3.52–6.69)	9.98(6.48–13.10)	7.11(3.94–10.50)	< 0.001
SUVpeak	Median (Q1, Q3)	3.27 (2.24–4.81)	7.06(5.14–9.75)	4.07(2.57–8.47)	< 0.001
MTV	Median (Q1, Q3)	0.75(0.33–1.82)	3.23(1.08–7.95)	1.22(0.51–3.32)	< 0.001
TLG	Median (Q1, Q3)	2.65(0.83–5.73)	23.86(8.89–51.36)	4.69(1.80-30.75)	< 0.001
Primary tumor size	Median (Q1, Q3)	16.0(13.0–23.0)	30.50(22.75-41.0)	20.0(16.0–39.0)	< 0.001

Abbreviations: SUVmax, maximum standardized uptake value; SUVpeak, peak standardized uptake value; TLG, total lesion glycolysis; MTV, metabolic tumor volume; FN, false-negative; TN, true-negative; TP, true-positive; FP, false-positive

## Discussion

With the widespread application of ¹⁸F-FDG PET/CT, researchers are increasingly focused on the association between metabolic characteristics and LNM in cancers [15, 23–25]. Although previous studies have confirmed the correlation between ¹⁸F-FDG PET/CT metabolic parameters and LNM in NSCLC [21, 26], the unique biological behavior of ALK-positive NSCLC characterized by a higher propensity for LNM deserves further targeted research. However, there are still few studies specifically evaluating the metabolic parameters of ^18^F-FDG PET/CT to predict the risk of LNM in ALK-positive NSCLC. This retrospective analysis aimed to address this gap by assessing the utility of ¹⁸F-FDG PET/CT metabolic parameters in stratifying LNM risk among ALK-positive NSCLC patients.

Accurate LNM staging is critical for optimizing treatment in ALK-positive NSCLC, as precise TKIs therapy based on staging can significantly improve progression-free survival and overall survival in this subgroup [27]. Moreover, controversies persist regarding the necessity of lymph node dissection in clinical T1 NSCLC [28–30], underscoring the need for reliable non-invasive biomarkers to guide surgical decisions. Consequently, validating ¹⁸F-FDG PET/CT as an effective tool for evaluating the risk of LNM in ALK-positive NSCLC could enable personalized treatment strategies, including timely TKIs therapy initiation and appropriate lymph node dissection.

In this study, we demonstrated that in the specific context of ALK-positive NSCLC, primary tumor size and SUVmax are the most robust independent predictors, a finding that refines the predictive model for this distinct molecular subtype. Secondly, and more importantly, we are the first to report that primary tumor metabolic burden, specifically TLG, can differentiate between true-negative and false-negative (OLNM) cases. This may could addresses a critical clinical challenge, which is that the high rate of unexpected nodal metastasis found postoperatively in ALK-positive patients despite negative preoperative ¹⁸F-FDG PET/CT reports. Our research offers a practical tool to preoperatively identify patients at high risk for OLNM. This finding has direct translational value, potentially guiding surgeons towards more extensive lymph node dissection in high-risk individuals, thereby improving the accuracy of initial treatment planning. Consistent with previous studies [31–33], all enrolled ALK-positive NSCLC patients had lung adenocarcinoma in this study. Notably, no significant differences in baseline characteristics, including age, BMI, gender, smoking history, or primary tumor location, were observed between patients with and without LNM. These findings potential suggested that LNM in ALK-positive NSCLC may be driven primarily by tumor-intrinsic biological factors rather than these traditional risk factors. The inability of conventional clinical parameters to stratify LNM risk in ALK-positive NSCLC further highlights the necessity of integrating quantitative ¹⁸F-FDG PET/CT metrics (e.g., SUVmax, TLG, and MTV) into nodal staging assessment. These parameters on ^18^F-FDG PET/CT demonstrated significant associations with LNM in our cohort and may better reflect underlying tumor aggressiveness, providing a robust foundation for clinical decision-making.

While ¹⁸F-FDG PET/CT metabolic parameters are established predictors of LNM in various cancers [15, 21–24, 26], their role in ALK-positive NSCLC remains underexplored. For instance, SUVmax have been linked to LNM in esophageal squamous cell carcinoma [16], and SUVpeak independently predicted LNM in general NSCLC cohorts [34]. Our study extends these findings by demonstrating significantly higher SUVmax, SUVpeak, MTV, and TLG in primary tumors of ALK-positive NSCLC patients with LNM versus those without. Univariate analysis confirmed markedly associations between all four parameters and LNM. However, multivariate analysis identified SUVmax as the sole independent predictor. This suggested that SUVmax may serve as a key biomarker for LNM risk stratification in ALK-positive NSCLC, potentially reflecting the unique metabolic activity of these tumors. Thus, ¹⁸F-FDG PET/CT parameters, particularly SUVmax, may serve as adjunctive tool to guide lymph node dissection or TKIs therapy in ALK-positive NSCLC patients, minimizing adverse outcomes from LNM misclassification.

OLNM remains a diagnostic challenge in NSCLC [13, 14]. Our study demonstrated that ^18^F-FDG PET/CT exhibits limited sensitivity (64.79%) and NPV (64.79%) for detecting LNM only based on status of lymph node in ALK-positive NSCLC patients, resulting in a high rate of false-negative (OLNM-positive), despite high specificity (96.51%) and PPV (93.9%). False-negative cases may be caused by metastatic lymph nodes that are too small, exhibit low metabolic uptake, or are too close to the primary tumor. We found that primary tumor metabolic parameters, including SUVmax, SUVpeak, TLG, MTV and tumor size, were significantly elevated in true-positive LNM cases compared to false-negative (OLNM-positive) and true-negative cases. Notably, OLNM patients demonstrated significantly larger tumor size and higher TLG than true-negative patients, while SUVmax, SUVpeak, and MTV lacked discriminatory power. This suggested that primary tumor TLG, a metric reflecting total metabolic burden, may serve as a critical biomarker to identify OLNM missed by ^18^F-FDG PET/CT.

We speculate that a high TLG reflects not only the size of the primary tumor but also an intrinsic, aggressive biological state driven by metabolic reprogramming. Tumors with high glycolytic activity, as quantified by TLG, typically exhibit a pronounced Warburg effect, which fuels rapid proliferation and facilitates the creation of a pro-invasive tumor microenvironment. This metabolic state is intrinsically linked to oncogenic pathways that drive epithelial-mesenchymal transition (EMT), thereby enhancing the capacity of tumor cells to detach and intravasate into lymphatic vessels. Consequently, a primary tumor displaying high metabolic burden is biologically primed for early dissemination and is more likely to have seeded regional lymph nodes with micrometastatic deposits. These microscopic clusters often remain undetected by conventional PET/CT due to limitations in spatial resolution and metabolic sensitivity, thus presenting as OLNM. We found that TLG rather than SUVmax was a key metabolic parameter, which strongly supports this hypothesis, because it underscores that the risk of occult transmission is driven by the total volume of biologically invasive tumors. Consequently, the integration of primary tumor TLG into clinical staging algorithm provides a promising strategy for reducing the risk of OLNM-related underestimation and guiding more accurate therapeutic decisions for ALK-positive NSCLC patients.

In addition, larger primary tumor size was significantly associated with LNM in ALK-positive NSCLC, which was consistent with previous studies that linked the tumor burden to the risk of LNM [13, 35–37]. Multivariate analysis further identified primary tumor size as an independent predictor of LNM in ALK-positive NSCLC patients, which was consistent with prior research linking larger tumors to higher LNM risk in ALK-positive NSCLC [7]. We found that prominent differences in primary tumor size were observed among false-negative (OLNM-positive), true-positive, and true-negative cases. When patients were stratified by ^18^F-FDG PET/CT diagnostic results, primary tumor size and TLG followed a stepwise pattern: true-positive > false-negative (OLNM) > true-negative (all pairwise *P* < 0.05). Notably, primary tumors in the FN group were larger than those in the TN group (median 20.0 mm vs. 16.0 mm; *P* = 0.031), suggesting that primary tumor size may help identify occult nodal disease that evades standard metabolic imaging. These data indicated that integrating primary tumor size with ^18^F-FDG PET/CT parameters could refine risk stratification for OLNM in ALK-positive NSCLC. Therefore, we believe it is necessary to conduct prospective studies to determine the optimal threshold of primary tumor size and TLG, and explore whether additional minimally invasive lymph node sampling should be considered for tumors exceeding this threshold, despite negative diagnostic results from ^18^F-FDG PET/CT.

This study has several limitations that should be acknowledged. First, this was a retrospective design inevitably introduces selection bias. Second, detailed analysis of ALK variants was not performed. We lacked data on specific ALK variants, which may have different metastatic behaviors, thus limiting the applicability of our results to all ALK-positive subtypes. Third, the single-center setting and modest sample size (*n* = 157) restrict generalizability. Fourth, the analysis focused solely on baseline ^18^F-FDG PET/CT parameters without longitudinal follow-up data, which limits assessment of the long-term prognostic value of these metabolic markers for LNM-related outcomes, such as recurrence-free survival or therapeutic response.

## Conclusions

In treatment-naïve ALK-positive NSCLC, SUVmax and tumor size of primary tumor are independent predictors of LNM risk, while TLG may help identify OLNM. Integrating these parameters into preoperative risk stratification can guide personalized lymph node dissection strategies and optimize ALK-TKIs sequencing decisions. Prospective trials should validate their utility in reducing unnecessary invasive procedures and improving survival outcomes in ALK-positive NSCLC.

## Data Availability

The data that support the findings of this study are available from the corresponding author, JZ, upon reasonable request.
